# A Dutch guideline for the treatment of scoliosis in neuromuscular disorders

**DOI:** 10.1186/1748-7161-3-14

**Published:** 2008-09-26

**Authors:** MG Mullender, NA Blom, M De Kleuver, JM Fock, WMGC Hitters, AMC Horemans, CJ Kalkman, JEH Pruijs, RR Timmer, PJ Titarsolej, NC Van Haasteren, MJ Van Tol-de Jager, AJ Van Vught, BJ Van Royen

**Affiliations:** 1 Dept. Orthopaedic Surgery, Vrije Universiteit Medical Center (VUmc), Research Institute MOVE, Amsterdam, The Netherlands; 2 Department of Pediatric Cardiology, Leiden University Medical Center, The Netherlands; 3 Dept. Orthopaedics, Sint Maartenskliniek, Nijmegen, The Netherlands; 4 Dept. Neurology, University Hospital Groningen, The Netherlands; 5 Revalidatiecentrum Blixembosch, Eindhoven, The Netherlands; 6 Vereniging Spierziekten Nederland, Baarn, The Netherlands; 7 Dept. Perioperative & Emergency Care, University Medical Centre Utrecht, The Netherlands; 8 Department of Paediatric Orthopaedics, University Hospital for Children and Youth Het Wilhelmina Kinderziekenhuis, Utrecht, The Netherlands; 9 Dept Anesthesiologie, University Hospital Maastricht, The Netherlands; 10 Groot Klimmendaal, Arnhem, The Netherlands; 11 Revalidatiecentrum Leijpark, Tilburg, The Netherlands; 12 Revalidatiecentrum de Hoogstraat en Wilhelmina Kinderziekenhuis UMC Utrecht, The Netherlands; 13 Department of Paediatrics, University Hospital for Children and Youth Het Wilhelmina Kinderziekenhuis, Utrecht, The Netherlands

## Abstract

**Background:**

Children with neuromuscular disorders with a progressive muscle weakness such as Duchenne Muscular Dystrophy and Spinal Muscular Atrophy frequently develop a progressive scoliosis. A severe scoliosis compromises respiratory function and makes sitting more difficult. Spinal surgery is considered the primary treatment option for correcting severe scoliosis in neuromuscular disorders. Surgery in this population requires a multidisciplinary approach, careful planning, dedicated surgical procedures, and specialized after care.

**Methods:**

The guideline is based on scientific evidence and expert opinions. A multidisciplinary working group representing experts from all relevant specialties performed the research. A literature search was conducted to collect scientific evidence in answer to specific questions posed by the working group. Literature was classified according to the level of evidence.

**Results:**

For most aspects of the treatment scientific evidence is scarce and only low level cohort studies were found. Nevertheless, a high degree of consensus was reached about the management of patients with scoliosis in neuromuscular disorders. This was translated into a set of recommendations, which are now officially accepted as a general guideline in the Netherlands.

**Conclusion:**

In order to optimize the treatment for scoliosis in neuromuscular disorders a Dutch guideline has been composed. This evidence-based, multidisciplinary guideline addresses conservative treatment, the preoperative, perioperative, and postoperative care of scoliosis in neuromuscular disorders.

## Introduction

Neuromuscular disorders comprise a very diverse group of disorders. The most common forms of neuromuscular disorders are Duchenne Muscular Dystrophy (DMD) of the myopathic disorders and Spinal Muscular Atrophy (SMA) of the neurogenic disorders. Despite differences in etiology both forms have a lot of characteristics in common. Both disorders have a genetic origin and have an effect on several systems, requiring a multidisciplinary evaluation and treatment. Typical for both of these disorders is the progressive character of the muscle weakness and as a result ventilatory restriction and a progressive scoliosis. Usually joint contractures, as well as nutritional disorders are present. In dystrophinopathy, cardiac dysfunction and mental retardation can occur as well.

Severe scoliosis causes discomfort and compromises respiratory function. Spinal surgery is considered the primary treatment option for correcting severe scoliosis in neuromuscular disorders. It can improve the cardio-pulmonary function, the sitting balance, appearance, and the quality of life. However, in this population surgery is considered a major intervention with high risk. Careful preoperative evaluation should not be restricted to the spinal deformity itself, but anesthetic management, pediatric cardio-pulmonary care, postoperative intensive care and rehabilitation should be emphasized. Several issues concerning the surgical intervention are still controversial. For instance, what is the best time point for surgery, and is surgery always needed? Due to these uncertainties, the VSCA, a cooperation of various institutions and patients' associations of patients on assisted ventilation, experienced some difficulties and inconsistencies in the management of scoliosis treatment in patients with DMD and SMA in the Netherlands. It was proposed that the management of scoliosis in neuromuscular disorders could benefit from clear guidelines and more uniformity in definitions, and terminology, amongst the various disciplines involved. The effectiveness and safety of scoliosis surgery in DMD patients was recently reviewed [1]. Due to the absence of randomized controlled clinical trials the authors recommended to refer to anecdotal evidence and expert opinions in order to guide decision making in scoliosis surgery. Hence, a multidisciplinary study group "guidelines scoliosis in neuromuscular disorders" started with the development of multidisciplinary guidelines based on the Dutch situation.

## Objectives

The objective of the Dutch guideline for the treatment of scoliosis in neuromuscular disorders is to present recommendations based on the evidence available in systematic reviews, randomized controlled trials, supplemented by cohort studies, and expert opinions. These recommendations are directed at a wide range of professionals dealing with the management of patients with scoliosis as a result of neuromuscular disease. The guideline strives for a multidisciplinary consensus about terminology, diagnostics, indications for surgery, surgical treatment, follow-up and patient education. Hence, the guideline supports a multidisciplinary approach and encourages collaboration between the different disciplines involved.

### Target population

The guideline is directed at professionals concerned with the management of scoliosis in patients with a neuromuscular disorder, and the professional associations that are involved in the treatment or care for these patients. Indirectly, these guidelines may inform relatives, and people otherwise engaged in the care for patients with neuromuscular disorders, educational organizations, and the general public.

### Guidelines working group

The guideline was developed by a multidisciplinary group of experts in the field of neuromuscular scoliosis management. All relevant specialties involved in diagnostics and treatment were represented. In addition, representatives from societies of interest participated in the working group in order to create a sound basis for the application of the guideline.

The working method employed consisted of: 1) identification of problems by the working group, 2) translation into specific questions, 3) search of scientific evidence by sub-groups in answer to the questions posed, 4) discussion of the evidence in the complete working group and 5) resolving the recommendations for best practice by the working group.

## Evidence

The guideline is based on evidence from the available scientific publications and expert opinions. The evidence was categorized according to its strength (Table 1). Consequently, the strength of the recommendations is graded based on the level of evidence (Table 2).

**Table 1:** Classification of evidence.

Papers concerning interventions (prevention or therapy)	
A1	Systematic reviews discussing at least a few studies of A2-level, and in which the results of the distinctive studies discussed are consistent;
A2	Randomized controlled trials (RCTs) of good quality (randomized double blind controlled trials) involving a sufficient number of subjects, and employing suitable research methods;
B	Generally consistent findings of weaker scientific studies (not randomized, comparative cohort studies, patient -control studies);
C	non-comparative studies;
D	expert opinions.
	
Papers concerning diagnosis	
A1	Prospective studies of the effects of diagnostics on clinical outcome in a well defined patient group. The outcome parameters are well defined in advance.
	Good quality studies of decision-making with respect to diagnostics and clinical outcomes;
A2	Studies of diagnostic tests compared to a reference test. Criteria and outcomes are well-defined in advance, and the test and the study population are adequately described. The study population must be sufficiently large. Independent observers have assessed the test results. The experimental and reference tests are evaluated independently. In cases where multiple diagnostic tests are used, the analysis should be adapted for interdependency of the results (e.g. by using logistic regression);
B	Studies of diagnostic tests compared to a reference test. The test and population are described but the study is of less quality than described in level A;
C	non-comparative studies;
D	expert opinions.

**Table 2:** Grading of the recommendations according to the level of evidence.

*Level 1*	At least one systematic review (A1) or at least 2 independent studies with evidence level A1 or A2
*Level 2*	At least two independent studies with evidence level B
*Level 3*	One study with evidence level A2 or B or multiple studies of level C
*Level 4*	Expert opinion

## Definitions

### Neuromuscular disorder

The general definition of a neuromuscular disorder is a disease pertaining to both the nerves and the muscles. However, the term appeared to be interpreted differently within the various disciplines. Hence, a more specific definition was proposed. In this guideline, a neuromuscular disorder is defined as a defective function of the peripheral nerve system, the neuromuscular junction, or muscles, causing muscle weakness in the patient. Disorders of the central nervous system, such as cerebral palsy or spina bifida, are not included in this definition. Within this definition, neuromuscular disorders are the result of defective anterior horn cell function in the spinal cord (e.g. SMA), defects of the peripheral nerves (neuropathies), the neuromuscular junction (myasthenia gravis), structural defects of the muscle cells (myopathies), muscle cell degradation (muscular dystrophies), or metabolic myopathies (glycogenosis, mitochondrial myopathy). Most of these disorders are rare. Therefore, the relatively frequent occurring progressive disorders DMD and SMA, both associated with a high incidence of scoliosis, were taken as paradigm for the treatment of neuromuscular scoliosis.

### Duchenne Muscular Dystrophy (DMD)

DMD is an X-linked (Xp21) recessive inherited disease. Affected males inherited the genetic mutation from the mother, or developed the condition by spontaneous mutations in the gene. Usually, the affected boy has a slightly delayed motor development during infancy. Hereafter, symptoms are a rapidly progressive muscle weakness associated with muscle wasting associated with a progressive difficulty to walk. Most boys are wheelchair-bound at the age of 10. Major changes during puberty are the progressive muscle weakness in hands, arms, and torso, associated with the development of scoliosis. In this phase, the respiratory function is gradually deteriorating, with need of respiratory assistance, and a possible cardiomyopathy may induce problems. Mild mental retardation is present in approximately 40% of DMD patients.

DMD is diagnosed by repeated determination of extremely elevated Creatine Kinase (CPK-MM) levels in the bloodstream and mutations in the dystrophin gene on the X-chromosome, or by the absence of the gene product dystrophin in a muscle biopsy.

### Spinal Muscular Atrophy (SMA)

SMA includes a number of genetic disorders, all presenting with a manifestation of weakness due to loss of the motor neurons of the spinal cord and brainstem. The most common form of SMA is caused by a mutation of the Survival Motor Neuron (SMN) gene, located on chromosome 5q13. It is an autosomal recessive form which manifests over a wide range of severity affecting infants through adults. The spectrum has been classified into four types based on the onset of the symptoms and the level of weakness.

*SMA type 1*, also known as severe infantile SMA or Werdnig Hoffmann disease, is the most severe, and manifests in the first year of life with the inability to ever maintain an independent sitting position. Children with SMA type 1 deteriorate rapidly and have a life expectancy of less than two years.

*SMA type 2*, or intermediate SMA, describes those children who are wheelchair-bound very early in life. Usually, they are never able to walk and a severe dystrophy associated with considerable scoliosis develops already around the age of four. Some of the children depend on respiratory assistance during the night, and later around the clock, with or without tracheostomy. The onset of weakness is usually recognized some time between 6 and 18 months. Within the first three years SMA types 1 and 2 cannot be distinguished based on determination of the genetic lesion. Consequently, this is a very uncertain period for the parents.

*SMA type 3*, also called juvenile SMA or Kugelberg-Welander disease has a more moderate course. Patients with SMA type 3 preserve their ability to walk until the second or third decade, and they develop less scoliosis and respiratory problems.

In *SMA type 4*, or adult SMA weakness usually begins in the third decade. The course of disease is much slower and has little or no impact on life expectancy.

This guideline is directed at the group of patients with SMA type 2.

SMA is diagnosed by the typical clinical symptoms and by the SMN gene test.

### Spinal deformation

Scoliosis is a complex deformation that involves abnormal lateral and rotational curvature of the spine. Both in DMD and SMA type 2 scoliotic deformations are frequent. The deformation is mostly a progressive thoracolumbar C-curve with development of pelvic obliquity. Either an increased kyphosis or thoracic lordosis can be part of the deformity. The consequences of the deformity are loss of sitting balance, shortening of the trunk, and compression of the hart and lungs. The mobility of the ribs is reduced by rotation and deformation of the trunk, causing obstruction of the breathing capacity.

## Epidemiology – incidence and prevalence of scoliosis

Epidemiologic data regarding scoliosis in DMD are provided by a few studies [2-5]. However, most studies are dated before the availability of DNA diagnostics. Possibly, Becker Muscular Dystrophy (BMD) is included in some of these study groups. This may be associated with a lower incidence of scoliosis reported in the earlier studies. Development of scoliosis in DMD patients is strongly related to the loss of walking ability. Generally, ambulatory patients do not develop a scoliosis. However, as the patient's walking ability deteriorates and patients become wheelchair dependent, scoliosis starts to develop, mostly in the thoracolumbar region. Neither severity nor rate of progression can be predicted from the age at which spinal collapse occurs or the age at which ambulatory ability is lost [3,5]. The incidence of spinal deformity in DMD varies between reports. No uniform method is used to describe the development of scoliosis. Oda et al. [3] define three types of spinal deformity, where type 3 is the type with the least deformity; a Cobb angle < 30°. They report that 15% (7/46) of patients who lost their walking ability belong to this group. Brooke et al. [2] reported that 75% of 120 patients developed a curve between 30° and 120°. In an early study of 60 DMD patients the incidence of scoliosis, defined as a curve between 5° and 120°, was 50% [4]. In a more recent study 76% of untreated patients (24) and 17% of patients treated with corticosteroids (30) developed a scoliosis with a curve > 20° [6].

All children with SMA type 2 develop scoliosis starting from an early age of around 3 years [7,8]. At ten years of age the average curve is more than 54° [8]. In 80% of the cases the scoliosis is a low thoracic curve. The single curve is most prevalent (83%), however 17% of the patients develop a double curve [8].

### Natural course of scoliosis

Data about the natural history of curve progression and the relationship between scoliosis, morbidity, and mortality are lacking. Although relationships have been suggested [2,3,9] such relationships have not been substantiated by data.

## 1. Conservative phase

### Factors affecting the progression of scoliosis in DMD

#### Evidence

Indications exist that not all patients with DMD develop a scoliosis. The natural course of scoliosis in DMD patients was studied by Oda et al. [3] and Yamashita et al. [10]. Oda et al. conclude that there is no indication for surgery when the Cobb's angle is less than 30° in 15 year old patients. This is the case in 15% of the described patient group. Yamashita et al. argue that in order to determine early which DMD patients need surgical intervention for treatment of spinal deformity, criteria should be developed to predict the progression of the deformity in the individual patient at an earlier age than 14 or 15 years. To predict which patients at the age of 10 are likely to develop a severe scoliosis they studied a group of 12 patients retrospectively and performed multiple discriminant analysis. The vital capacity (VC), and Cobb angle of spinal scoliosis at the age of 10 years, the age at which ambulation ceased, and the curve pattern of spinal scoliosis were found to be predictors for the progression of scoliosis. They propose a model based on these predictors to guide the judgment whether surgical stabilization of the spine should be considered.

#### Discussion/conclusion

In the absence of DNA verification it is unsure if all patients in the studies of Oda et al. [3] and Yamashita et al. [10] are DMD patients. Possibly, patients with BMD were included as well. Hence, the percentage of DMD patients that do not develop a scoliosis remains uncertain. Nevertheless, it is plausible that surgery to correct spinal scoliosis is not indicated in all DMD patients *(level 2) *[3,10]. The criteria suggested by Yamashita et al. predicting the progression of scoliosis may still be relevant and could be applied to patients with a diagnosis of DMD. The decline in pulmonary function is presumably correlated with the progression of scoliosis *(level 2) *[3,10]. The decline in vital capacity is, however, expected to occur as part of the natural history of the condition in patients with DMD and/or SMA and this is not necessarily causally related to the development of scoliosis. It is imperative that the diagnosis of DMD is confirmed by DNA analysis. This may help to uncover which proportion of established DMD patients does not develop a progressive scoliosis.

#### Recommendation

▪ It is important to find out whether a child with DMD belongs to the small minority that does not develop a severe scoliosis. For this purpose, the respiratory function should be monitored in children with DMD, since the vital capacity is a possible indicator of the progression of scoliosis.

### Factors affecting the progression of scoliosis in SMA type 2

#### Evidence

Progressive spinal deformity occurs in all children with SMA type 2 (workgroup opinion (WO)).

#### Discussion/conclusion

The incidence of scoliosis in SMA type 2 is 100%. Hence, all children with SMA type 2 are at some time indicated for surgical intervention *(level 4)*.

#### Recommendation

▪ All children with SMA type 2 should be operated to correct the spinal deformity at some time point.

### Conservative treatment of scoliosis in DMD

#### Evidence

Brace treatment and sitting orthoses cannot prevent the development or progression of scoliosis in DMD patients [11,12]. Some findings suggest that prolongation of walking by the use of orthoses can prevent a rapid progression of scoliosis in DMD patients between 13 and 15 years of age [11,13]. However, Bakker et al. [14] concluded in a review of 35 studies that it is uncertain whether knee-ankle-foot orthoses can prolong functional walking, although it seems that the use of knee-ankle foot orthoses can prolong assisted walking and standing.

There is no evidence that physical training can influence the evolution of muscular dystrophies in the long term. If the patient rejects surgical intervention, orthotic treatment or seating adaptations build on the patients' wheelchair can be beneficial to improve the sitting comfort and to prevent decubitus.

#### Discussion/conclusion

Findings in literature and experience from experts indicate that orthoses do not prevent the development or progression of scoliosis in DMD *(level 3) *[11,12]. Therefore, orthoses should not be recommended for this purpose. Rodillo et al. [13] presented evidence that prolongation of the walking ability by the use of long leg braces may prevent the rapid progression of scoliosis in DMD patients between 13 and 15 years of age *(level 3)*. However, these findings have not been corroborated since. In addition, it is uncertain if patients who maintained their walking ability until the age of 13 did not have a milder form of DMD. In addition, Bakker et al. [13] concluded that it is uncertain if knee-ankle-foot orthoses can prolong functional walking. It is concluded that the evidence is not sufficient to recommend the use of long leg braces to prevent scoliosis in DMD patients.

#### Recommendation

▪ Orthoses are not recommended to prevent the development or progression of scoliosis in DMD patients.

▪ For patients who reject surgery sitting-orthoses can be considered for improvement of sitting comfort.

### Conservative treatment of scoliosis in SMA type 2

#### Evidence

Development of scoliosis in patients with SMA type 2 is inevitable. Orthopedic treatment with a spinal brace or long leg braces does not affect the course of scoliosis development [15,16]. There are no data suggesting that orthoses in general influence scoliosis progression in SMA type 2 patients.

It is experienced in practice, however, that spinal braces to improve comfort and sitting balance are appreciated by patients with SMA type 2 in the preoperative phase. On the other hand, rigid spinal braces were shown to have a negative influence on the respiratory function [17].

#### Discussion/conclusion

Spinal braces and sitting orthoses do not prevent the development or progression of scoliosis in SMA type 2 *(level 3) *[15]. Walking with long leg braces cannot prevent the development or progression of scoliosis in SMA type 2 *(level 3) *[16]. A rigid spinal brace has a negative effect on respiratory function *(level 3) *[17].

#### Recommendation

▪ Orthoses are not recommended to prevent the development or progression of scoliosis in SMA type 2 patients.

▪ In the preoperative phase a non rigid spinal brace or sitting orthosis can be considered for the improvement of comfort and sitting balance.

### Steroid treatment of scoliosis in DMD

#### Evidence

A few studies indicate that corticosteroids may delay the development and progression of scoliosis in DMD patients [6,18,19]. In a non-randomized comparative study, it was found that a smaller percentage of DMD patients treated with Deflazacort (a prednisolon derivative) developed scoliosis and that progression of scoliosis was less severe compared to patients that did not receive corticosteroids [6].

#### Discussion/conclusion

A significant effect of long term treatment with corticosteroids on the prevalence and progression of scoliosis was found *(level 2) *[6,18,19]. Hence, treatment with corticostreroids may be considered for this purpose. A Dutch guideline is already available for the use of corticosteroids in DMD patients [20]. This guideline recommends the use of prednisone in relative low doses, which reduces the chance of side-effects, including osteopenia or osteoporosis.

#### Recommendation

▪ Treatment with corticosteroids to prevent the development or progression of scoliosis in DMD patients may be considered, even if the patient is wheelchair-bound.

▪ It is recommended to follow the Dutch guidelines [20] for the usage of corticosteroids in DMD patients.

### Contractures in DMD

#### Evidence

In a retrospective study of 45 DMD patients, Furderer et al. [21] describe a positive correlation between spinal convexity and hip contracture. However, causal relationships were not established. In a retrospective study of 54 patients Chan et al. [22] found that there was a correlation between migration percentage of the femoral head and scoliosis. They suggested that scoliosis potentiated the development of hip subluxation and dislocation in DMD.

#### Discussion/conclusion

Although a correlation has been established between spinal convexity and hip contracture [21,22], no indications can be found in literature that contractures in the lower extremities contribute to the development or progression of scoliosis *(level 3)*. Based on retrospective data, Chan [22] even suggests a reverse causality, i.e. scoliosis potentiates the development of hip subluxation. However, no studies are available using X-rays of pelvis and spine to follow the development of scoliosis and contractures in young DMD patients.

#### Recommendation

▪ Corrective surgery of lower extremities in order to prevent the development or progression of scoliosis is not recommended in DMD patients.

### Contractures in SMA type 2

#### Evidence

Granata [23] studied 49 SMA patients, of which 35 were type 2. He described a linear correlation between migration percentage of the hip and scoliosis. However, no causal relationship between contractures in the lower extremities and scoliosis was established.

#### Discussion/conclusion

Based on a linear correlation between age and migration percentage, and between migration percentage and degree of scoliosis, Granata et al. suggested that hip subluxation and scoliosis develop simultaneously due to muscle weakness around the hip [23]. In their study, they also show that the pattern of scoliosis does not match the type of pelvic obliquity in a consistent way. This indicates that contractures in the lower extremities do not govern the development of scoliosis and supports their suggestion that muscle weakness is the common cause of both hip subluxation and scoliosis *(level 3)*.

#### Recommendation

▪ No evidence exists for a causal relationship between contractures in the lower extremities and scoliosis. Hence, corrective surgery of lower extremities in order to prevent the development or progression of scoliosis is not recommended in SMA type 2.

## 2. Preoperative phase

### General

In the preoperative phase the respiratory and cardiac functions should be assessed according to an explicit protocol in relation to both the timing of surgery and possible post-operative complications. Unfortunately, available evidence about these factors in DMD and SMA type 2 patients is limited. All studies concerning the course of spinal surgery in DMD and SMA type 2 patients are non-comparative retrospective studies. Some studies suggest guidelines for preoperative protocols.

### Preoperative protocol respiratory function

#### Evidence

The relationship between deterioration of pulmonary function and the occurrence of postoperative complications is uncertain. Some indications are given that preoperative pulmonary function is an important factor based on experience and small cohort studies [24,25]. However, few studies were found in which the relationship between pulmonary function and postoperative complications was quantified [26-28]. Jenkins et al. [27] correlated preoperative respiratory function tests with postoperative respiratory complications in 48 DMD patients. They found that the percentage of predicted vital capacity provided the best indicator of outcome, and values of less than 30% were associated with major respiratory complications. Yet, in another study no different complication rates were found comparing DMD patients with a preoperative forced vital capacity (FVC) of more than 30% and patients with a vital capacity of less than 30% after spinal surgery [26,28]. They concluded that spinal fusion surgery can be offered to patients with DMD even in the presence of a low vital capacity [26,28]. Gill at al. [29] studied a small group of patients with progressive scoliosis and rare forms of muscular dystrophy/myopathy with respiratory failure. They concluded that patients with preexisting respiratory failure on nocturnal noninvasive ventilation can be safely operated for deformity correction [29].

Several studies have evaluated the effect of spinal stabilization in DMD patients on the rate of decline in respiratory function [9,27,30-33], but the results are at variance. Some studies comparing operated with non-operated patients found no difference in rate of decline [9,30,33]. Yet others found that spinal fusion reduced the rate of respiratory decline [27,31,32].

No literature is available about the benefit of preoperative practice with non-invasive ventilation in patients with neuromuscular scoliosis. However, it is the opinion of the workgroup that it may be beneficial to practice with noninvasive ventilation in patients with FVC ≤ 40% of the predicted value and/or indicated by arterial blood gas analysis.

#### Discussion/conclusion

The relationship between preoperative pulmonary function and postoperative complications is indistinct *(level 3) *[9,26,26-30,33]. The traditionally accepted lower limit of vital capacity to allow spinal surgery in DMD patients is not validated by evidence *(level 3) *[27,31,32]. Spinal fusion may reduce the rate of decline in respiratory function.

#### Recommendation

▪ Respiratory function should be tested in all patients with neuromuscular scoliosis.

▪ In case of seriously impaired pulmonary function (FVC ≤ 40% of predicted value) it is advised to practice preoperatively with noninvasive ventilation.

▪ In case of seriously impaired pulmonary function (FVC ≤ 40% of predicted value) it is advised to teach patients techniques, such as air stacking, which can assist patients with their sputum evacuation.

### Preoperative protocol cardiac function

#### Evidence

Literature about cardiac dysfunction with regard to spine surgery in neuromuscular diseases is scarce. Cardiac dysfunction, as a result of cardiomyopathy, typically occurs in patients with DMD and seldom in patients with SMA type 2. The risk of surgery is greatly increased by cardiac dysfunction or rhythm abnormalities [34]. Therefore preoperative medical therapy for cardiac dysfunction in patients with muscular dystrophy is required. The cardiomyopathy of DMD is characterized by fibrosis of the posterobasal and contiguous lateral wall of the left ventricle, and is associated with arrhythmia, ventricular dilatation and cardiac insufficiency.

#### Discussion/conclusion

Neuromuscular disorders, especially DMD, often involve cardiac problems in the course of the disease, such as a reduced contractility and arrhytmia. Therefore a preoperative consultation at the pediatric cardiologist (with echocardiography (ECG) and, in case of arrhythmia, a 24 hour holter ECG) is required for DMD patients (ACC/AHA guidline 2002) [35]. It is the opinion of the workgroup that severe cardiac dysfunction in DMD may be an indication for early spinal surgery or a contraindication for surgery *(level 4)*.

#### Recommendation

▪ The pediatric cardiologist should be consulted during the preoperative planning of surgery in DMD patients.

### Preoperative protocol other considerations

#### Evidence

The usefulness of multidisciplinary preoperative protocols has never been studied. However, it is generally agreed that the preoperative planning of spinal surgery in neuromuscular scoliosis requires a multidisciplinary approach.

#### Discussion/conclusion

Based on expert opinions it is recommended that attention is given to nutrition, bowel and bladder function, contractures, blood coagulation, medication, and postoperative care, besides pulmonary and cardiac function *(level 4) *[36]. Furthermore, several issues should be discussed preoperatively with the parents. These include the possibility of postoperative ventilation (with tracheostomy in particular) and resuscitation decision making. The severity of cardiac and pulmonary problems can be a reason to discard surgery.

#### Recommendation

▪ Attention should be given to nutrition, bowel and bladder function, contractures, blood coagulation, medication, and postoperative care during the preoperative phase.

▪ Preoperatively, resuscitation decision making should be discussed with the parents.

▪ Preoperatively, the possible requirement of postoperative ventilation (tracheostomy) should be discussed with the parents.

### Anesthetic considerations

#### Evidence

No studies were found specifically addressing anesthesia in SMA type 2 or DMD patients, but useful information can be obtained from several mixed series of patients with neuromuscular disorders treated according to explicit guidelines [24,25,37-39]. Best practice for perioperative blood management and spinal cord monitoring can be obtained from general literature about spine surgery. Hence, all recommendations are based on expert opinions only. Recommended procedures are hypotensive anaesthesia, the use of cell salvage and Tranexamic acid, hemodilution, hemodynamic monitoring, and careful positioning of a nasogastric tube and (Foley catheter or) indwelling urinary tract catheter. In addition, it is important to maintain normothermia in order to prevent hemorrhagic complications and clotting problems. The use of anticholinergica should be avoided.

#### Discussion/conclusion

Invasive hemodynamic monitoring, normvolemic hemodilution to reduce exposure to homologous bloodproducts, insertion of a nasogastric tube and (Foley catheter or) indwelling urinary tract catheter, careful maintenance of normothermia are described as expert opinions in literature *(level 4) *[25,37-39].

#### Recommendation

Peri-operative monitoring of patients with neuromuscular disorders should comply with the standard guidelines for major spine surgery.

## 3. Operative treatment

### Spinal fixation and fusion

#### Evidence

Posterior segmental fixation (in particular pedicle screw fixation or sublaminar wires) was found to be superior to distraction spondylodesis with Harrington rod instrumentation, since it gives better preservation of correction, less rod failure, and does not require postoperative bracing [40-42]. Anterior release and/or instrumentation and fusion in DMD and SMA is not advised [43]. With respect to the fusion length, spondylodesis up to Th4 or Th5 resulted in a higher rate of late deformity compared to instrumentation to Th3 or higher [42]. Pelvic fixation did not prove to be superior to instrumenting to L5 in DMD patients [44]: fixation to S1 improves the possibilities for correction of severe curves, but it is associated with loss of function, increases the duration of surgery and blood loss, and increases the complication rate. Fusion to L5 in neuromuscular scoliosis proved to be equivalent to fusion to the pelvis and the presence of a mobile lumbosacral joint may assist in seating and transfer activities [45].

The use of allograft bone for posterior fusion reduces operation time and blood loss compared to the use of autologous bone graft with similar clinical outcomes [46]. No studies are available on bone substitutes.

Early surgery is favored in DMD patients based on the progression of the curve (at 12 years the Cobb's angle was 20° versus a Cobb's angle of 50° at 14 years) [40,47]. No literature is available about SMA patients under the age of ten. If fusion is performed on these young children, the growth of the trunk should be taken into account to prevent torsion of the spine (crankshaft). The use of growth-sparing instrumentation can be considered in patients who have progressive spinal deformity and significant spinal growth remaining. Luque 'trolleys' have been used to control progressive curves using segmental instrumentation. The use of dual growing-rod technique in DMD and SMA type 2 has not been reported up to now.

#### Discussion/conclusion

Several aspects of spinal fixation in neuromuscular scoliosis have been evaluated. In DMD patients early surgery is favored *(level 3) *[40,47]. Posterior segmental instrumentation and fusion with the use of allograft bone combined with locally harvested bone is advocated in neuromuscular scoliosis *(level 3) *[40-43,46]. Correction of scoliosis and instrumentation should be at least from Th2 or Th3 *(level 3) *[42]. The use of dual growing-rod technique has not been reported up to now, however, repeated lengthening every 6 months may induce significant risks (serial anaesthetics, instrumentation problems, wound infections) *(level 4)*. Pelvic fixation gives better possibilities to correct severe curves, but increases time of surgery and blood loss *(level 3) *[44,47-49]. All studies are retrospective cohort studies (level C, D) and hampered by selection bias, however, the findings are consistent with the experience of the work group members. The SMA patients tend to fall into 2 groups surgically: (1) those who develop a significant scoliosis aged 1–3 years, and (2) those who develop a curve at the age of 4–7 years. The first group can be well managed with lengthening systems. In the second group, the curve usually remains flexible enough to delay surgery until the age of 7–9. At this age, although a spinal fusion will result in some loss of final sitting height and probably chest volume, the benefits of a single surgical procedure outweigh the risks of multiple lengthening procedures. The use of multiple pedicle screws around the curve apex may reduce the risk of crank-shafting *(level 4)*.

#### Recommendation

▪ The indication for surgical correction of scoliosis should be made early in DMD en SMA type 2 patients (Cobbs angle 20°), such that surgery is less complicated, shorter, and safer and it is more likely that the pelvis can be left out of the fusion trajectory.

▪ In DMD, posterior spinal fusion should be performed from high thoracic (Th2-3) at least to L4 or L5. Pelvic fixation gives better possibilities to correct severe curves, but increases time of surgery and blood loss.

▪ In SMA type 2 posterior spinal fusion should be performed. The technique varies with age and should consider possible remaining growth.

▪ Instrumentation should be segmental.

▪ Allograft bone can be used supplemented with locally harvested autologous bone or bone substitutes.

### Spinal cord monitoring

#### Evidence

The value of spinal cord monitoring for prevention of neurologic deficits during scoliosis surgery has generally been acknowledged [50]. In nine studies spinal cord monitoring in neuromuscular scoliosis has been described: the use of somatosensory evoked potential (SSEP) as a method for monitoring was assessed in seven [51-57], intraoperative transcranial electrical motor evoked potential (TCE-MEP) monitoring was evaluated in two studies [58,59]. The SSEP technique showed impermissible high variance if only one recording site was used [52], however, if the SSEPs were recorded from multiple sites located cortically, subcortically, and peripherally reliable values are obtained in 95–100% of the patients [51,53-57]. The more recent TCE-MEP method was described in a pilot study with 9 patients [59] and compared to SSEP in a prospective descriptive study including a group of 29 neuromuscular scoliosis patients and a group of 39 patients with cerebral palsy scoliosis [58]. It was concluded that both transcranial electric motor and posterior tibial nerve somatosensory-evoked potentials can be monitored reliably in most patients with neuromuscular scoliosis. Advantages of TCE-MEP relative to SSEP are the ability to test motor function during surgery and the possibility to easily adapt the recording sites to the remaining motor function.

#### Discussion/conclusion

Pre-operative neurological function varies considerably between patients with neuromuscular scoliosis and differs from healthy subjects. However, also for these patients it is important to prevent any clinical damage to the spinal cord to assure preservation of the remaining neurological functions. Spinal cord monitoring has shown to be applicable also in neuromuscular scoliosis *(level 3) *[51,53-59]. The methods of SSEP monitoring have been improved by the use of multiple recording sites, TCE-MEP allows for direct testing of important motoric functions.

#### Recommendation

▪ Spinal cord monitoring is recommended for neuromuscular scoliosis surgery. Both SSEP and TCE-MEP can be monitored reliably.

### Post-operative care

#### Evidence

Patients with DMD and SMA type 2 have a high risk of postoperative complications. The most frequent postoperative problems are related to the limited respiratory capacity of the patients. Cardiac dysfunction is rarely a cause for postoperative ventilation. No literature could be found about the postoperative treatment of DMD or SMA type 2 patients. The duration of postoperative ventilation is determined individually and is less than 36 hours in most cases [26,27]. The guidelines for placement in the pediatric intensive care unit (PICU) of the Dutch Pediatric Society are: ventilation >24 hours (children > 1 year) or failure of more than one organ system.

#### Discussion/conclusion

All statements are based on expert opinions *(level *4). Frequent use of non-invasive ventilation following extubation and cough assist machines in patients with an ineffective cough, intensive respiratory physical therapy, as well as early mobilisation of the patients on a reclining wheelchair may reduce the risk of postoperative pulmonary infection. The workgroup follows the guidelines of the Dutch Pediatric Society with respect to pediatric intensive care. Hence, only children without expected cardiac or pulmonary problems can be operated in a center without a pediatric intensive care unit.

#### Recommendation

▪ Only neuromuscular scoliosis patients older than 1 year, without respiratory complaints, without nightly hypoventilation, without cardiomyopathy requiring medication, with adequate pulmonary function, and where no adverse events are expected during surgery may be operated in a center without a pediatric intensive care unit. This is in accordance with the guidelines of the Dutch Pediatric Society.

### Therapeutic effect of scoliosis surgery on cardiac and pulmonary function in DMD

#### Evidence

All evidence is based on retrospective cohort studies (level 3). The effect of scoliosis surgery on cardiac function has not been studied. Comparison between pre- and post operative pulmonary function reveals no improvement due to correction of scoliosis. Long term studies show that decline of pulmonary function in DMD patients is unchanged in operated patients relative to patients who had no surgery [9,30,60]. Galasko et al. [32] reported a temporary halt in the decline of pulmonary function after scoliosis surgery, however after 2 years follow-up, the results were comparable to those reported by the other authors. Eagle et al. [61] found in a retrospective cohort study of 100 DMD patients that survival was improved in patients who received nocturnal ventilation and further improved in patients who received spinal surgery, however, due to the study setup no strong conclusions can be drawn.

#### Discussion/conclusion

Decline in pulmonary function in DMD patients is not influenced by scoliosis surgery *(level 3) *[9,30,32,60].

#### Recommendation

▪ When considering spinal surgery for DMD patients, the effects of surgery on heart and lungs do not play a role in the decision making.

### Therapeutic effects of scoliosis surgery in SMA type 2

#### Evidence

A few retrospective cohort studies reported the effects of spinal surgery on cardiac and pulmonary function in SMA type 2 patients. Most studies evaluate a mixed group of SMA patients. In a few cases improvement of the FVC after spinal surgery was reported [41,43].

#### Discussion/conclusion

In theory, spinal surgery could lead to improvement of pulmonary function, since stabilization of the spine stabilizes the chest cavity resulting in a reduction of the intra-abdominal pressure. Early intervention could thereby enable a better development of pulmonary function. However, presently not enough evidence exists to support this hypothesis *(level 3)*.

#### Recommendation

▪ Early scoliosis surgery may be considered although there is no evidence that spinal surgery decelerates the deterioration of cardiac and/or pulmonary function.

### Therapeutic effects of scoliosis surgery on activities of daily living (ADL)

#### Evidence

All studies are retrospective cohort studies and no comparison is made between treatment and natural course. Studies with long term follow-up all mention an improved sitting balance and subjective quality of live [41,43,62-64]. Brown [41] and Furumasu [64] reported improvement of several functions, however, other functions such as eating and personal care worsened.

#### Discussion/conclusion

Sitting balance is an important function for patients with neuromuscular scoliosis. Scoliosis surgery can improve sitting balance *(level 4) *[41,43,62-64]. The results are dependent on the surgical technique and the time of surgery. However it has adverse effects on some arm functions (eating, personal care). The decline of arm function is specifically seen after scoliosis surgery with trunk lengthening.

#### Recommendation

▪ Early scoliosis surgery is recommended in order to obtain a good and lasting improvement of the sitting balance. Education about the alteration of the trunk position and associated changes in arm function is necessary.

## 4. Precare and aftercare

### Information

#### Evidence

No scientific research is available about informing patients and parents about spine surgery in neuromuscular scoliosis. However, the importance of adequate information has been emphasized [65,66] and is endorsed by the work group.

#### Discussion/conclusion

The surgical techniques in neuromuscular scoliosis are similar to those in scoliosis surgery in patients without neuromuscular disease. However, the complexity of multiple physical handicaps demands specific pre-operative and peri-operative care. In two Dutch hospitals information brochures were available. Parents and patients should be adequately informed about the pre-operative examinations, surgery, post-operative care, risks, and consequences of the surgery *(level 4)*. Both rehabilitation specialist and orthopedic surgeon should inform the parents and the patient about these issues, possibly in a joint consult.

#### Recommendation

▪ Pre-operative preparation, surgery and after-care should be given by an experienced multidisciplinary team of specialists.

▪ Patients and parents should be well informed about all aspects of the treatment and the possible adverse events, including the aims of the surgery and the consequences for daily functioning (such as reduced arm function).

### Patients with physical and mental disability

#### Evidence

No evidence is available.

#### Discussion/conclusion

The prevalence of mental retardation in DMD is approximately 40%. SMA type 2 and mental retardation are not related. Pre- and postoperative care and information should be adjusted to the mental capacity of the patient *(level 4)*.

#### Recommendation

▪ In patients with a mental handicap information should be attuned.

### Postoperative care and aftercare

#### Evidence

An average hospital stay of 14 days was reported by Benson et al. [37]. Patients were mobilize in a wheel chair from day 5. Cotton [67] states that special care should be taken to prevent infection of the airways. He reports that mobilization in a wheelchair takes place from day 2. This is in agreement with other literature. No evidence was found about aftercare.

#### Discussion/conclusion

Patients are usually mobilized in a wheelchair from day 2–5. Nursing care comprises prevention of airway infections and decubitus, wound care, nutrition, and pain management *(level 3) *[37,67].

In the absence of evidence the work group has formulated their expert opinions on this subject *(level 4)*:

• Care is more intensive and complex during the first period after discharge from the hospital because children are weakened temporarily by the surgery and the healing process of the bony fusion takes 3–9 months. Rotation in the spine and hip flexion exceeding 90° are not allowed in this period. This has consequences for turning and lifting of the patients. A mobile hoist for the task of transferring a patient should be used. Before discharge the physical therapist should give instructions about lifting and sitting upright.

• In consultation with the general practitioner temporary homecare may be arranged.

• Measures should be taken to take care of changes in arm function due to the lengthening of the spine.

• For the recovery period at home, homework assignments can be provided.

• Because of the altered sitting position, the wheelchair needs to be adapted. Care should be taken that the procedures to realize these changes are started timely. The ergotherapist supervises this.

• The wheelchair should be adapted to optimize sitting comfort.

#### Recommendation

▪ After deciding on scoliosis surgery, the orthopedic surgeon and rehabilitation specialist should anticipate possible post-operative (temporary) problems and alterations in required care.

▪ The (electric) wheelchair should be adapted in order to support a position in which sitting balance and head balance can be maintained with the least effort for the child.

### Postoperative bracing

#### Evidence

No comparative studies are available about post-operative bracing. Large variation exists in policies regarding the use of post-operative braces. Braces are often not prescribed, but if prescribed most often synthetic braces are used for a post-operative period of 3 months [63,68-72].

#### Discussion/conclusion

Policies regarding post-operative bracing vary considerably (level 3). The workgroup comments that with the current instrumentation techniques braces are redundant and can be unfavorable for respiratory recovery, especially at the early stage following surgery when pulmonary function is significantly reduced.

#### Recommendation

▪ Post-operative spinal braces are not recommended with the modern instrumentation techniques.

### Head and Neck

#### Evidence

No literature could be found about the effectiveness of rehabilitation treatment after scoliosis surgery to prevent obliqueness of the head/neck.

#### Discussion/conclusion

No evidence is available concerning the post-operative treatment of head and neck posture. It is observed that sometimes a scoliotic deformation remains above the fusion trajectory. This should be taken into account when adaptations are made to the wheelchair. Possibly a head support is needed.

#### Recommendation

▪ Attention should be given to any changes in head and neck balance after scoliosis surgery. Possibly extra provisions or adaptations of the wheelchair may be needed.

### Management

#### Evidence

No studies have been conducted about the management of neuromuscular scoliosis.

#### Discussion/conclusion

Recommendations are all based on experience and opinions of experts within the workgroup *(level 4)*.

In the pre-operative phase, orthopedic surgeon and rehabilitation specialist preferably inform the parents and patients about the surgery, care, and consequences. The pediatrician, center for ventilation at home, and/or pulmonary specialist have to be consulted for screening of the pulmonary function. In DMD patients, the cardiologist has to be consulted for possible cardiomyopathy. Pre-operative screening must be performed as described above.

During surgery the anaesthesiologist considers all specific aspects of the neuromuscular disease (as described above). After surgery the patient is admitted at the intensive care unit. A threatening cardiomyopathy or respitory insufficiency requires monitoring at the pediatric intensive care. A physiotherapist coaches the child with respiratory techniques and coughing.

After surgery, the orthopedic surgeon has regular follow-ups and checks the sitting position in the wheelchair. The rehabilitation specialist verifies if adequate care is provided at home and coordinates adaptation of facilities.

#### Recommendation

▪ Spine surgery in neuromuscular scoliosis requires a multidisciplinary team of specialist experienced with treatment of these patients. Close cooperation is needed all specialists involved, including physiotherapist and nursing staff.

▪ The use of a checklist for treatment and post-operative care of spine surgery in neuromuscular scoliosis is advised.

## Abbreviations

BMD: Becker Muscular Dystrophy; DMD: Duchenne Muscular Dystrophy; ECG: Echocardiography; PICU: Pediatric intensive care unit; RCT: Randomized controlled trials; SMA: Spinal Muscular Atrophy; SMN: Survival Motor Neuron; SSEP: Somatosensory evoked potential; TCE-MEP: Transcranial electrical motor evoked potential; VC: Vital capacity; VSCA: Dutch cooperation of various institutions and patients' associations of patients on assisted ventilation; WO: Workgroup opinion.

## Competing interests

The authors declare that they have no competing interests.

## Authors' contributions

MGM wrote the manuscript together with BJVR, who was the chair of the working group. All other authors were members of the working group. They participated in the literature research and the formulation of the guide line. All authors read and approved the final manuscript.
